# Data on cytotoxicity in HeLa and SU-DHL-4 cells exposed to DPB162-AE compound

**DOI:** 10.1016/j.dib.2017.03.034

**Published:** 2017-03-23

**Authors:** Mart Bittremieux, Katsuhiko Mikoshiba, Geert Bultynck

**Affiliations:** a KU Leuven, Laboratory of Molecular and Cellular Signaling, Department of Cellular and Molecular Medicine & Leuven Kanker Instituut, 3000 Leuven, Belgium; b The Laboratory for Developmental Neurobiology, Brain Science Institute, RIKEN, 2-1 Hirosawa, Wako, Saitama 351-0198, Japan

**Keywords:** DPB162-AE, Cell toxicity, Cell lines, Calcium

## Abstract

DPB162-AE is a valuable tool to study store-operated Ca^2+^ entry (SOCE), as this compound was developed as a 2-APB analog that inhibits SOCE more potently and more selectively than 2-APB itself. In addition to this, we showed that, in some conditions, DPB162-AE can deplete the endoplasmic reticulum Ca^2+^ stores in intact cells, including the cervical carcinoma HeLa cell line and the diffuse large B-cell lymphoma SU-DHL-4 cell line. Here, we present data regarding the toxicity of DPB162-AE in HeLa and SU-DHL-4 cells. For further interpretation of the data presented in this article, please see the research article ‘DPB162-AE, an inhibitor of store-operated Ca^2+^ entry, can deplete the endoplasmic reticulum Ca^2+^ store’ (M. Bittremieux, J. V. Gerasimenko, M. Schuermans, T. Luyten, E. Stapleton, K.J. Alzayady, et al., 2017) [1].

**Specifications Table**

**Table:** 

Subject area	*Biology*
More specific subject area	*Ca*^*2+*^*signaling*
Type of data	*Graphs*
How data was acquired	*CellTox*^*™*^*Green Cytotoxicity Assay (Promega). Fluorescence was read on a FlexStation 3 microplate reader (Molecular Devices).*
Data format	*Analyzed*
Experimental factors	*HeLa and SU-DHL-4 cells were stained with the CellTox*^*TM*^*Green dye and treated with different concentrations of DPB162-AE (1, 3, 10 and 30 µM), vehicle (DMSO) as negative control and lysis solution as positive control condition. Cells were incubated at 37 °C and 5% CO*_*2*_.
Experimental features	*Cytotoxicity was determined by measuring the fluorescence intensity of the CellTox*^*™*^*Green dye after treatment of the cells with DPB162-AE for different time periods.*
Data source location	*KU Leuven, Leuven, Belgium*
Data accessibility	*All data are presented in this article.*

**Value of the data**

• The data show the potential cytotoxic effect of DPB162-AE in HeLa and SU-DHL-4 cells.
• The data indicate that DBP162-AE is not toxic to HeLa and SU-DHL-4 cells up to concentrations of 3 μM when applied for 24 h, while it is toxic to SU-DHL-4 cells for concentrations of 10 μM and higher for time periods of about 16 h and longer.
• The data highlight differences in cytotoxic sensitivity between cell lines for DPB162-AE.
• These data may be relevant for (i) other researchers using DPB162-AE in their experiments and (ii) for further research that focuses on the impact of SOCE inhibition accompanied by sustained ER Ca^2+^-store depletion for cell survival.
• A protocol is provided to easily screen compounds for their cytotoxic effects using the CellTox^™^ Green Cytotoxicity assay.

## 1 Data

**Fig. 1:**
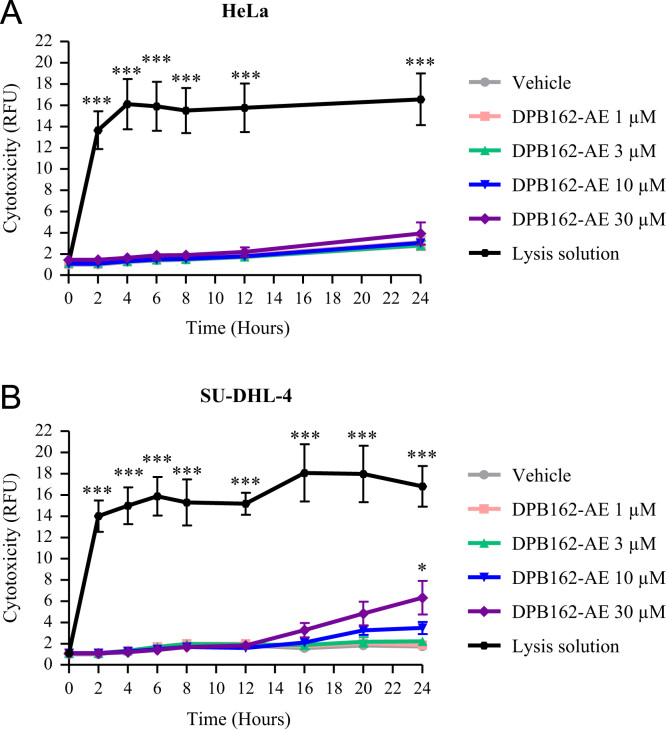
The cytotoxic effect of DPB162-AE on (A) HeLa and (B) SU-DHL-4 cells. Cells were treated with 1, 3, 10 and 30 µM of DPB162-AE. Vehicle (DMSO) was used as negative control, while lysis solution was used as positive control condition. Cell death (RFU) was measured after 2, 4, 6, 8, 12, 16, 20 and 24 h of treatment. All values were first corrected for the background control. Data were normalized to the values obtained for the vehicle-treated condition at time point zero, which was set at 1. Data are represented as mean±SEM of at least three independent experiments.

In this report, we present data on the cytotoxicity of DPB162-AE, which is an inhibitor of store-operated Ca^2+^ entry that can also deplete the ER Ca^2+^ stores [1], [2], in two cell lines, i.e. adherent HeLa cells and non-adherent SU-DHL-4 cells. In both cell lines, cytotoxicity was determined by measuring the fluorescence intensity of the CellTox^™^ Green dye. This fluorescence intensity correlates with the loss of cell membrane integrity occurring as a result of cell death. The raw data values of the HeLa and SU-DHL-4 cells are shown in Table 1, Table 2 respectively, whereas the normalized data obtained after subtracting the background control are depicted in Fig. 1. DPB162-AE was not toxic for HeLa cells, since they were almost completely resistant to DPB162-AE concentrations up to 30 µM applied for 24 h (Fig. 1A). In contrast, SU-DHL-4 cells were more sensitive to DPB162-AE when applied for prolonged time periods. After 16 h of treatment, 10 and 30 µM of DPB162-AE induced toxicity in SU-DHL-4, whereas lower concentrations of DPB162-AE did not trigger cell death in this cell line (Fig. 1B).

## 2 Experimental design, materials and methods

### 2.1 Cell culture

Diffuse large B-cell lymphoma SU-DHL-4 cells were cultured at 37 °C and 5% CO_2_ in suspension in RPMI-1640 medium (Invitrogen). Human cervical carcinoma HeLa cells were cultured at 37 °C and 5% CO_2_ in DMEM medium (Invitrogen). All media were supplemented with 10% heat-inactivated FBS, L-glutamine and penicillin and streptomycin. Both cell lines have been authenticated using autosomal STR profiling performed by the University of Arizona Genetics Core and fully matched the DNA fingerprint present in reference databases.

### 2.2 Cytotoxicity assay

Cell death induced by DPB162-AE was determined in HeLa and SU-DHL-4 cells using the CellTox^™^ Green Cytotoxicity assay (Promega), in which cell death is measured with a fluorescent dye that binds the DNA of cells with impaired membrane integrity. This cell death assay allows user-friendly kinetic cytotoxicity measurements in culture, since the same well can be measured multiple times as the fluorescent signal remains constant after 72 h of exposure. HeLa cells were seeded in a 96-well plate (Greiner) at a density of 15,000 cells/well. SU-DHL-4 cells were seeded in a 96-well plate with poly-L-lysine coating at a density of 250,000 cells/well. Next, a mixture of CellTox^™^ Green Dye and cell medium (1:1000) was added to the 96-well plate. Subsequently, cells were treated with different concentrations of DPB162-AE (1, 3, 10 and 30 µM), vehicle (DMSO) as negative control condition and lysis solution as a positive control. Wells without cells were used as a background control. Cell death was determined after 2, 4, 6, 8, 12, 16, 20 and 24 h of treatment by measuring the fluorescence intensity (485/520 nm excitation/emission) with a FlexStation 3 microplate reader (Molecular Devices). The values obtained from the background control were first subtracted from the values obtained from the different experimental conditions. These data were normalized to the values obtained for the vehicle-treated condition at time point zero, which was set at 1.

### 2.3 Statistical analysis

Results are reported as mean±SEM of at least three independent experiments. In each independent experiment three technical replicates were used. Significance was determined using a one-way ANOVA with a post-hoc Dunnett׳s multiple comparison test versus vehicle-treated cells. Differences were considered significant at *p*<0.05.

## Figures and Tables

**Table 1:** Raw data values of CellTox^™^ Green fluorescence in HeLa cells. Cells were treated with 1, 3, 10 and 30 µM of DPB162-AE. Vehicle (DMSO) was used as negative control, while lysis solution was used as positive control condition. A background control was used to normalize the raw data values. Cell death (RFU) was measured before treatment (0 h) and after 2, 4, 6, 8, 12 and 24 h of treatment. The raw data values of 4 independent experiments are shown as mean±SD. Each independent experiment consisted of 3 technical replicates. The average values of the independent experiments are shown as mean±SEM.

**HeLa**			**CellTox Green fluorescence (RFU)**						
**Treatment**			**0 h**	**2 h**	**4 h**	**6 h**	**8 h**	**12 h**	**24 h**
**Vehicle**	Exp. 1	Mean±SD	185.1±8,8	193.1±30,4	228.0±39,3	232.8±42,4	244.7±34,3	269.6±37,2	370.9±40,1
	Exp. 2		58.5±0,5	69.5±8,4	66.8±5,5	76.5±14,8	71.3±11,6	76.8±14,7	80.3±13,0
	Exp. 3		99.6±3,8	109.6±5,8	130.9±7,8	133.2±7,6	144.1±4,5	166.8±16,9	280.3±45,9
	Exp. 4		119.1±5,1	126.8±2,2	146.4±10,3	152.3±12,3	160.6±13,6	184.2±11,5	295.8±24,5
	*Average*	*Mean*±*SEM*	*115.6*±*26,3*	*124.8*±*25,7*	*143.0*±*33,1*	*148.7*±*32,3*	*155.2*±*35,5*	*174.4*±*39,5*	*256.8*±*62,0*
**DPB162-AE 1 µM**	Exp. 1	Mean±SD	196.5±12,0	210.9±3,5	249.5±6,1	252.5±13,5	274.4±17,2	302.0±16,5	403.6±18,7
	Exp. 2		56.6±1,3	68.9±5,4	67.0±6,7	76.0±10,3	70.2±5,1	76.1±6,9	84.0±9,2
	Exp. 3		104.7±5,4	128.2±15,3	154.1±13,5	160.6±12,4	166.8±16,9	200.0±18,5	328.8±20,2
	Exp. 4		96.2±19,4	111.4±12,7	134.9±11,6	141.9±11,2	150.6±10,3	184.7±6,2	322.1±34,6
	*Average*	*Mean*±*SEM*	*113.5*±*29,5*	*129.9*±*29,7*	*151.4*±*37,6*	*157.8*±*36,4*	*165.5*±*41,9*	*190.7*±*46,2*	*284.6*±*69,3*
**DPB162-AE 3 µM**	Exp. 1	Mean±SD	190.1±8,9	200.6±6,9	244.5±4,2	251.8±5,4	277.3±6,8	299.6±12,0	397.2±20,6
	Exp. 2		64.5±1,4	78.7±4,6	70.5±1,9	79.4±2,4	74.0±3,7	76.6±2,8	83.6±2,0
	Exp. 3		102.7±2,0	118.6±3,4	136.4±7,9	144.7±3,5	147.2±5,5	173.3±10,7	290.6±8,8
	Exp. 4		107.1±4,0	121.2±12,0	142.7±9,9	149.8±12,5	155.3±11,2	176.2±13,3	289.2±20,3
	*Average*	*Mean*±*SEM*	*116.1*±*26,4*	*129.8*±*25,5*	*148.5*±*35,9*	*156.4*±*35,6*	*163.5*±*42,1*	*181.4*±*45,6*	*265.2*±*65,5*
**DPB162-AE 10 µM**	Exp. 1	Mean±SD	199.1±14,0	204.6±34,6	245.0±47,0	257.0±37,1	282.7±36,4	308.0±33,6	423.3±29,0
	Exp. 2		59.9±2,9	77.3±3,9	72.8±4,9	83.9±7,3	79.9±3,0	84.8±2,3	105.9±6,3
	Exp. 3		95.6±2,0	110.9±14,3	135.0±16,7	138.6±13,8	144.1±12,2	166.0±13,1	285.1±17,9
	Exp. 4		114.9±6,0	120.0±8,1	140.9±7,9	144.3±5,0	151.2±14,8	162.4±7,4	263.5±17,3
	*Average*	*Mean*±*SEM*	*117.4*±*29,5*	*128.2*±*27,0*	*148.4*±*35,6*	*156.0*±*36,3*	*164.5*±*42,5*	*180.3*±*46,5*	*269.5*±*64,9*
**DPB162-AE 30 µM**	Exp. 1	Mean±SD	196.4±5,3	214.2±16,9	253.9±12,3	260.2±16,5	278.8±17,8	292.7±12,3	386.9±33,7
	Exp. 2		63.8±3,0	102.8±7,2	98.9±8,3	115.1±9,3	111.2±12,0	122.6±13,4	204.9±40,2
	Exp. 3		107.0±2,6	119.9±3,9	141.1±3,7	146.2±3,5	147.9±1,0	164.6±2,4	263.6±12,9
	Exp. 4		113.6±22,7	130.8±21,8	145.2±19,4	150.2±19,1	149.7±21,0	175.9±28,1	257.0±33,0
	*Average*	*Mean*±*SEM*	*120.2*±*27,6*	*141.9*±*24,7*	*159.8*±*33,0*	*167.9*±*31,7*	*171.9*±*36,7*	*189.0*±*36,4*	*278.1*±*38,5*
**Lysis solution**	Exp. 1	Mean±SD	195.1±8,8	1387.9±82,2	1532.5±81,1	1516.3±96,0	1511.7±99,8	1504.9±93,3	1558.8±104,7
	Exp. 2		67.5±3,0	1242.5±71,3	1091.4±125,9	1295.5±148,6	1254.8±74,2	1256.2±77,3	1217.6±79,1
	Exp. 3		104.0±7,0	1156.2±29,7	1379.2±31,4	1357.9±51,5	1312.0±47,6	1346.2±48,2	1413.9±62,5
	Exp. 4		129.5±19,0	1072.9±83,0	1294.1±104,2	1282.7±133,1	1245.9±136,1	1267.8±142,0	1338.0±164,8
	*Average*	*Mean*±*SEM*	*124.0*±*26,8*	*1214.9*±*67,2*	*1324.3*±*91,9*	*1363.1*±*53,6*	*1331.1*±*61,9*	*1343.8*±*57,3*	*1382.1*±*71,4*
**Background control**	Exp. 1	Mean±SD	74.0±0,6	76.9±2,4	76.8±2,9	76.8±2,9	76.8±2,9	76.8±2,9	72.3±1,6
	Exp. 2		45.1±1,7	46.5±0,4	43.7±0,3	44.8±0,3	42.7±1,0	42.7±0,7	42.1±1,7
	Exp. 3		47.3±0,2	48.4±0,2	46.5±0,6	43.5±0,8	44.9±0,6	48.8±1,7	47.5±0,8
	Exp. 4		44.3±1,4	44.7±0,6	44.1±1,1	43.3±0,6	42.8±0,8	42.1±0,2	42.8±1,2
	*Average*	*Mean*±*SEM*	*52.7*±*7,1*	*54.1*±*7,6*	*52.8*±*8,0*	*52.1*±*8,2*	*51.8*±*8,3*	*52.6*±*8,2*	*51.2*±*7,1*

**Table 2:** Raw data values of CellTox^™^ Green fluorescence in SU-DHL-4 cells. Cells were treated with 1, 3, 10 and 30 µM of DPB162-AE. Vehicle (DMSO) was used as negative control, while lysis solution was used as positive control condition. A background control was used to normalize the raw data values. Cell death (RFU) was measured before treatment (0 h) and after 2, 4, 6, 8, 12, 16, 20 and 24 h of treatment. The raw data values of 3 independent experiments are shown as mean±SD. Each independent experiment consisted of 3 technical replicates. The average values of the independent experiments are shown as mean±SEM.

**SU-DHL-4**			**CellTox Green fluorescence (RFU)**								
**Treatment**			**0 h**	**2 h**	**4 h**	**6 h**	**8 h**	**12 h**	**16 h**	**20 h**	**24 h**
**Vehicle**	Exp. 1	Mean±SD	125,5±17,4	128.6±11,4	174.6±10,0	149.5±25,1	204.6±5,0	215.2±15,0	172.0±6,7	182.1±8,7	222.6±20,4
	Exp. 2		169.8±9,8	184,0±17,4	205,4±12,2	269,6±18,4	272,2±20,7	270,7±18,3	228,0±11,6	287,8±26,5	214,7±8,3
	Exp. 3		124.7±8,9	132,0±21,2	198.6±49,9	261.2±53,8	245,2±57,5	250,1±82,5	201.9±24,9	229.2±34,0	330,9±42,4
	*Average*	*Mean*±*SEM*	*140.0*±*14,9*	*148.2*±*17,9*	*192.9*±*9,3*	*226.8*±*38,7*	*240.7*±*19,6*	*245.3*±*16,2*	*200.7*±*16,1*	*233.0*±*30,5*	*256.1*±*37,4*
**DPB162-AE 1 µM**	Exp. 1	Mean±SD	142.2±28,7	143,7±10,2	171,9±15,0	200,3±18,3	214,1±21,6	223,7±21,3	184,1±7,1	201,3±1,6	232,9±18,2
	Exp. 2		165.5±16,1	187,4±7,5	197,0±4,7	248,8±14,4	282,4±8,8	273,3±0,2	266,2±24,7	329,8±31,8	247,8±12,9
	Exp. 3		150.8±6,6	158,2±11,5	216,1±33,3	271,9±40,3	294,1±45,2	296,1±78,7	226.1±49,9	250.5±46,8	321,4±14,6
	*Average*	*Mean*±*SEM*	*152.8*±*6,8*	*163.1*±*12,8*	*195.0*±*12,8*	*240.3*±*21,1*	*263.5*±*24,9*	*264.4*±*21,3*	*225.5*±*23,7*	*260.5*±*37,4*	*267.4*±*27,3*
**DPB162-AE 3 µM**	Exp. 1	Mean±SD	139.3±22,1	146,0±8,3	184,9±5,8	196,0±12,2	219,7±15,2	217,2±11,8	173,7±2,9	183,5±3,4	226,2±5,5
	Exp. 2		169.2±14,9	181,6±13,9	186,8±10,2	233,0±9,6	259,6±13,4	248,1±16,0	265,5±15,5	345,7±45,0	294,3±35,9
	Exp. 3		153.3±7,9	155,7±11,4	206,1±18,2	267,3±30,7	296,0±46,1	294,3±51,9	261.3±48,5	292.1±35,3	318,2±32,7
	*Average*	*Mean*±*SEM*	*153.9*±*8,6*	*161.1*±*10,6*	*192.6*±*6,7*	*232.1*±*20,5*	*258.4*±*22,0*	*253.2*±*22,4*	*233.5*±*29,9*	*273.8*±*47,7*	*279.6*±*27,5*
**DPB162-AE 10 µM**	Exp. 1	Mean±SD	128.1±16,5	131,2±14,5	159,8±11,2	148,3±7,5	160,2±9,2	170,9±12,3	189,4±3,2	283,0±1,9	400,6±49,9
	Exp. 2		163.7±26,1	186,4±23,2	189,7±20,4	227,8±12,4	249,5±22,1	237,9±21,3	374,8±13,5	618,5±40,3	453,1±55,7
	Exp. 3		147.7±2,6	170,7±32,4	206,6±26,8	251,9±26,4	270,9±20,3	246,2±23,4	254.8±30,0	356.4±39,1	309,5±12,7
	*Average*	*Mean*±*SEM*	*146.5*±*10,2*	*162.8*±*16,4*	*185.4*±*13,6*	*209.3*±*31,3*	*226.9*±*33,9*	*218.3*±*23,8*	*273.0*±*54,2*	*419.3*±*101,8*	*387.7*±*41,9*
**DPB162-AE 30 µM**	Exp. 1	Mean±SD	124,7±24,6	127,3±13,0	154,0±20,1	149,7±12,8	174,9±17,0	228,2±17,7	268,0±8,1	350,8±6,3	470,7±42,8
	Exp. 2		186,2±7,8	187,8±4,8	221,8±11,9	253,5±8,6	268,6±14,2	290,8±8,6	513,2±48,6	787,6±97,6	543,7±136,8
	Exp. 3		157.6±8,7	169,2±18,3	192,8±23,9	239,2±25,0	260,2±43,7	241,9±32,5	429.3±53,2	637.3±42,8	1014.0±57,7
	*Average*	*Mean*±*SEM*	*156.2*±*17,7*	*161.4*±*17,8*	*189.5*±*19,6*	*214.1*±*32,4*	*234.6*±*29,9*	*253.6*±*19,0*	*403.5*±*71,9*	*591.9*±*128,1*	*676.1*±*170,2*
**Lysis solution**	Exp. 1	Mean±SD	129.3±29,8	1784.0±150,8	1851.2±202,4	1759.0±182,5	2019.0±209,0	1786.0±115,3	2233.0±230,1	2272.0±237,9	1694.0±287,7
	Exp. 2		168.4±29,1	2571.1±363,7	2490.1±318,7	2772.0±403,8	2796.0±205,7	2752.0±407,9	2062.0±51,2	2138.0±50,5	2838.0±415,1
	Exp. 3		142,0±15,2	1021.3±222,3	1301.5±255,1	1441.0±223,3	1432.0±325,9	1378.0±509,7	2360.5±251,1	2316.9±256,2	1482.0±602,1
	*Average*	*Mean*±*SEM*	*146.6*±*11,5*	*1792.1*±*447,5*	*1881.0*±*343,6*	*1991.0*±*401,3*	*2082.0*±*395,0*	*1972.0*±*407,4*	*2218.0*±*86,4*	*2242.0*±*53,7*	*2005.0*±*421,1*
**Background control**	Exp. 1	Mean±SD	18.4±0,4	18.6±0,9	18.2±0,5	17.9±0,9	18.5±1,2	18.4±1,3	18.3±0,6	18.7±0,3	18.4±1,0
	Exp. 2		16.2±0,4	16.7±0,6	15.9±0,2	16.7±0,3	16.7±0,5	16.6±0,7	16.6±0,7	16.7±0,4	16.7±0,4
	Exp. 3		26.6±1,0	28.8±1,0	28.0±0,9	28.0±0,3	27.5±0,6	28.2±0,7	18.7±0,6	17.7±0,2	28.4±0,3
	*Average*	*Mean*±*SEM*	*20.4*±*3,1*	*21.4*±*3,7*	*20.7*±*3,7*	*20.9*±*3,5*	*20.9*±*3,3*	*21.1*±*3,5*	*17.9*±*0,6*	*17.7*±*0,5*	*21.2*±*3,6*
