# Supplementary material for: Data on cytotoxicity in HeLa and SU-DHL-4 cells exposed to DPB162-AE compound
Source: Data Brief. 2017 Mar 23;12:91–6. doi: 10.1016/j.dib.2017.03.034 (PMC5377240; doi:10.1016/j.dib.2017.03.034)
Supplement: Supplementary file 1 — Supplementary material [file mmc1.docx]

The authors declare that there is no conflict of interest related to this work.
